# Occurrence of bornaviruses, circoviruses and polyomaviruses in necropsy samples from parrots in Poland (2014–2024)

**DOI:** 10.2478/jvetres-2026-0011

**Published:** 2026-03-25

**Authors:** Ines Szotowska, Aleksandra Ledwoń

**Affiliations:** Department of Pathology and Veterinary Diagnostics, Warsaw University of Life Sciences, 02-776, Warsaw, Poland

**Keywords:** avian polyomavirus, beak and feather disease virus, circovirus, proventricular dilatation disease, psittacine

## Abstract

**Introduction:**

Proventricular dilatation disease (PDD) is a disease of parrots which has been known for many years, but only in 2008 was it confirmed that its cause is avian bornavirus (ABV). Further disease aetiology and epidemiology information came subsequently. Later research distinguished an eight-genotype group of parrot bornaviruses (PaBV) infecting psittacines. In addition to PaBV, circoviruses (beak and feather disease viruses – BFDV) and avian polyomaviruses (APyV) also represent major viral pathogens of parrots. These may occur concurrently. This study’s intent was to determine the occurrence of PaBV, BFDV and APyV in parrots in Poland between 2014 and 2024 and to compare it with extranational data.

**Material and Methods:**

Samples taken from 210 naturally deceased breeder-owned and pet parrots necropsied between 2014 and 2024 were studied. Tissue samples were analysed by RT-PCR (PaBV) and nested PCR (BFDV and APyV).

**Results:**

Parrot bornavirus RNA was detected in 50 parrots (23.8%), BFDV DNA in 59 (28%), and APyV DNA in 65 parrots (31%). A dilated proventriculus and gizzard – changes typical for PDD – were found in 22 (10.5%) cases and 18 were confirmed by a positive RT-PCR result. Thirty-two (15.2%) birds tested positive for PaBV despite the absence of obvious PDD lesions.

**Conclusion:**

Bornavirus infections in parrots in Poland occur relatively often, but less frequently than infections with polyomaviruses and circoviruses. Only 36% of the birds with confirmed bornavirus infections had changes typical for PDD observed in necropsy.

## Introduction

Viral diseases remain a major health concern in companion and aviary parrots (14), with three pathogen groups being of particular relevance: beak and feather disease virus (BFDV), avian polyomavirus (APyV) and avian bornaviruses (ABVs). These agents differ in genetic structure and pathogenic potential – BFDV and APyV are DNA viruses (8, 16), whereas ABVs are RNA viruses (11, 18) – but all may circulate silently within flocks (8, 11, 16, 18).

Clinical signs associated with these infections vary. Beak and feather disease virus causes chronic and fatal psittacine beak and feather disease associated with feather dystrophy, beak deformities and immunodeficiency (8, 16). Avian polyomavirus infection is especially problematic in budgerigars, where it leads to budgerigar fledgling disease and feather abnormalities, but is also damaging to breeding facilities where it can be a cause of chick deaths (8). Avian bornaviruses cause proventricular dilatation disease (PDD), bornavirus. characterised clinically by weight loss, regurgitation, undigested seeds in faeces, diarrhoea and neurological dysfunction (6). Parrot bornaviruses (PaBVs) comprise eight known genotypes (PaBV-1 to -8) (3). Transmission likely occurs *via* damaged mucosa or skin, with subsequent spread along nerves to the spinal cord, brain, enteric ganglia, heart and kidneys (1). Vertical transmission has also been proven (12).

The aim of this study was to determine the occurrence of PaBV, BFDV and APyV in necropsy samples from parrots in Poland between 2014 and 2024 and to compare our findings with epidemiological data from other countries.

## Material and Methods

### Samples

A total of 210 naturally deceased parrots of 43 species were submitted for necropsy by 55 owners between 2014 and 2024. The birds were single individuals from households and birds from large groups in breeder aviaries. For each bird, species, origin and housing type were recorded.

### Necropsy

Standard examinations were performed. Samples of the brain, liver, spleen, kidneys and lungs were collected for molecular testing. Organs showing gross abnormalities were additionally described. Dilatation of the proventriculus and gizzard was evaluated subjectively based on macroscopic enlargement, thinning of the walls and retention of ingesta. No quantitative measurements were performed.

### RNA and DNA isolation

Genetic material was extracted from approximately 25 mg of pooled tissue using commercially available kits (Total RNA Mini Plus Kit and Genomic Mini AX Tissue Spin Kit, A&A Biotechnology, Gdańsk, Poland) according to manufacturer protocols. Extracted nucleic acids were stored at –33°C to –20°C until analysis.

### Detection of parrot bornaviruses

Screening for PaBV was performed using a OneStep RT-PCR (EURx, Gdańsk, Poland) following the protocol of Kistler *et al*. (9). The primer pair of ABV_NconsensusF and ABV_NconsensusR amplified an approximately 350 bp fragment of the viral N gene. Amplicons were purified and Sanger sequenced bidirectionally (Genomed, Warsaw, Poland). Chromatograms were inspected visually to ensure sequence quality using FinchTV software v.1.4.0 (Geospiza, now Revvity Signals, Waltham, MA, USA).

### Phylogenetic analysis

Analysis of genetic sequences of bornavirus isolates were performed using MEGA v. 12 software (10). For phylogenetic analysis, partial N gene nucleotide sequences were aligned by multiple sequence comparison by log-expectation. Genetic distances were then calculated using the Jukes–Cantor model and the tree was built by the unweighted pair-group method with arithmetic mean. Reference sequences for PaBV genotypes 1-5, 7 and 8 and Borna disease virus 1 (BoDV-1) were retrieved from GenBank. Parrot bornavirus 6 was excluded because only partial M-gene sequences are available. Support for phylogenies was measured by bootstrapping 1,000 replicates (2).

### Detection of circovirus (BFDV) and polyomavirus

Reactions for BFDV and APyV DNA were carried out using the nested PCR protocols described by Halami *et al*. (5) and Johne *et al*. (7), respectively. All assays included positive and negative controls.

## Results

### Frequency of viral infections

**Fig. 1.**
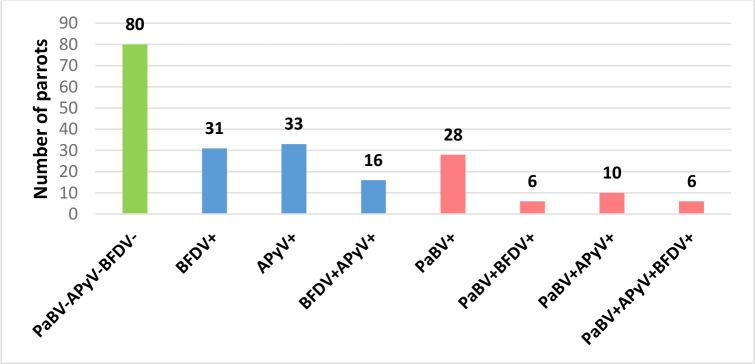
Infection patterns among necropsied parrots in Poland 2014–2024, including absence of tested viruses (green), single and mixed infections without parrot bornavirus (PaBV) (blue), and infections involving PaBV (pink). BFDV – beak and feather disease virus; ApyV – avian polyomavirus

Viral infections were common, with at least one of the three tested viruses (PaBV, BFDV and APyV) detected in 130 birds (61.9%) (Fig. 1). Parrot bornavirus RNA was identified in 50 parrots (23.8%), BFDV DNA in 59 parrots (28%), and APyV DNA in 65 parrots (31.0%). Mixed infections were observed in 38 birds (18.1%), among them co-infections involving PaBV were noted in 22 individuals (10.5%).

### Necropsy findings

Typical macroscopic lesions consistent with PDD, including marked dilatation or thinning of the proventriculus or gizzard and retention of ingesta, were observed in 22 parrots (10.5%). Of these, 18 tested positive for PaBV (36% of PaBV-positive birds). Conversely, 32 PaBV-positive parrots (64%) showed no gross lesions suggestive of PDD.

### Genotyping and phylogenetic analysis of PaBV

**Fig. 2.**
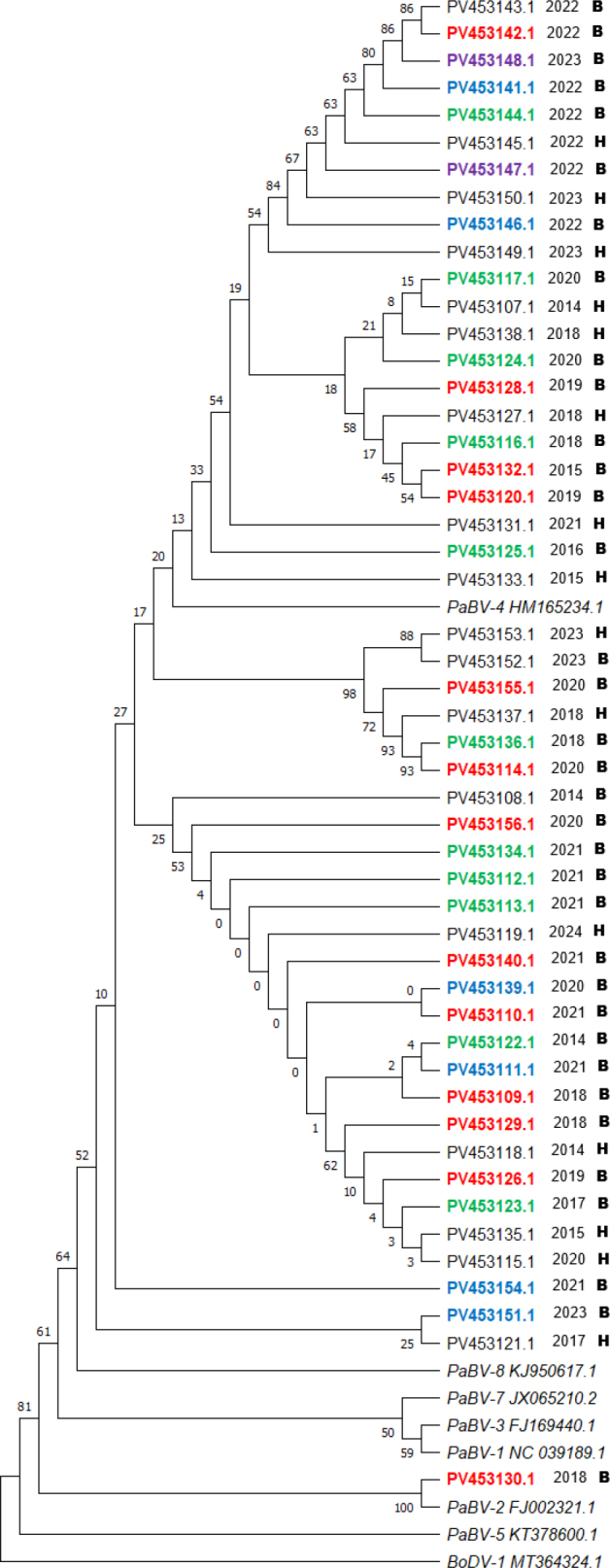
Phylogenetic relationships between parrot bornavirus (PaBV) strains detected in tissue samples of naturally deceased parrots in Poland 2014–2024. The tree is based on partial N-gene sequences and was constructed with distances calculated with the Jukes–Cantor model and using the unweighted pair-group method with arithmetic mean. Bootstrap values (1,000 replicates) are shown at the nodes. Reference sequences of PaBV-1 to PaBV-5, PaBV-7 and PaBV-8 and Borna disease virus 1 (BoDV-1; outgroup) were retrieved from GenBank; PaBV-6 could not be included due to absence of N-gene sequences in public databases. Two-letter and seven-digit codes are GenBank accession numbers. Black accession numbers – all samples origin from birds from different owners; coloured accession numbers – sequences written in one colour origin from the same owner; B – breeder aviary; H – home birds (from one to a few individuals); BoDV – Borna disease virus

Sequencing was successful for all 50 PaBV-positive samples, and the resulting sequences were included in the phylogenetic analysis. All but one samples clustered within PaBV-4, confirming that this genotype is strongly dominant among parrots in Poland. A single sequence (PV453130.1 taken in 2018 from a breeder-aviary bird) grouped within PaBV-2, representing an isolated introduction without evidence of further spread. The results of the phylogenetic analysis are presented as a phylogenetic tree (Fig. 2).

### Yearly distribution of infections

Infections with PaBV, BFDV and APyV occurred throughout the study period, though their frequencies varied significantly between years. The highest proportions of PaBV-positive parrots were recorded in 2020 and 2023. Circovirus and polyomavirus infections showed a more even annual distribution, with APyV being the most frequently detected virus overall. Detailed data are shown in Table 1.

**Table 1.** Yearly distribution of infections

Year	Total number of birds tested	PaBV-positive birds	BFDV-positive birds	APyV-positive birds
2014	10	4 (40.00%)	2 (20.00%)	5 (50.00%)
2015	6	3 (50.00%)	0 (0.00%)	4 (66.67%)
2016	9	1 (11.11%)	2 (22.22%)	6 (66.67%)
2017	16	3 (18.75%)	12 (75.00%)	6 (37.50%)
2018	18	9 (50.00%)	6 (33.33%)	6 (33.33%)
2019	30	3 (10.00%)	12 (40.00%)	5 (16.67%)
2020	34	5 (14.71%)	11 (32.35%)	11 (32.35%)
2021	26	8 (30.77%)	9 (34.62%)	9 (34.62%)
2022	29	7 (24.14%)	2 (6.90%)	3 (10.34%)
2023	15	6 (40.00%)	2 (13.33%)	3 (20.00%)
2024	17	1 (5.88%)	1 (5.88%)	7 (41.18%)

1 PaBV – parrot bornavirus; BFDV – beak and feather disease virus; ApyV – avian polyomavirus

### Species distribution of infections

Susceptibility to each virus varied between parrot groups. Infections with PaBV were most commonly detected in macaws (*Ara* and *Diopsittaca* spp.), amazons (*Amazona* spp.) and caiques(*Pionites* spp.). Budgerigars (*Melopsittacus undulatus*) – the most frequently submitted species – showed high infection rates for APyV and BFDV but infrequent PaBV positivity. Data are presented in Table 2.

**Table 2.** Species distribution of infections

Species	Scientific name	Total number of birds tested	PaBV-positive birds	BFDV-positive birds
cockatiel	*Nymphicus hollandicus*	21	3	1
Solomons corella	*Cacatua ducorpsii*	1	1	0
Pink cockatoo	*Cacatua leadbeateri*	0	0	0
grey parrot	*Psittacus erithacus erithacus*	3	1	1
Senegal parrot	*Poicephalus senegalus*	3	1	1
monk parakeet	*Myiopsitta monachus*	3	1	0
blue-headed parrot	*Pionus menstruus*	1	0	1
blue-fronted amazon	*Amazona aestiva*	17	4	6
yellow-headed amazon	*Amazona oratrix*	4	0	0
orange-winged amazon	*Amazona amazonica*	1	0	0
festive amazon	*Amazona festiva*	1	0	1
Cuban amazon	*Amazona leucocephala*	1	1	0
barred parakeet	*Bolborhynchus lineola*	3	1	1
caique	*Pionites* spp.	11	3	1
green-cheeked parakeet	*Pyrrhura molinae*	5	2	1
burrowing parrot	*Cyanoliseus patagonus*	1	0	1
sun conure	*Aratinga solstitialis*	2	2	0
military macaw	*Ara militaris*	1	0	0
golden-collared macaw	*Primolius auricollis*	1	0	0
blue-winged macaw	*Primolius maracana*	3	0	0
hyacinth macaw	*Anodorhynchus hyacinthinus*	1	0	1
chestnut-fronted macaw	*Ara severus*	2	1	0
blue-and-yellow macaw	*Ara ararauna*	7	5	3
Ara harlequin	*Ara ararauna* × *chloroptera*	1	1	0
red-and-green macaw	*Ara chloroptera*	2	1	0
scarlet macaw	*Ara macao*	1	0	0
red-shouldered macaw	*Diopsittaca nobilis*	4	4	2
regent parrot	*Polytelis anthopeplus*	3	0	0
red-breasted parakeet	*Psittacula alexandri*	3	0	0
plum-headed parakeet	*Psittacula cyanocephala*	5	2	4
rose-ringed parakeet	*Psittacula krameri*	8	1	2
Alexandrine parakeet	*Psittacula eupatria*	1	1	0
Bourke’s parrot	*Neopsephotus bourkii*	2	2	0
scarlet-chested parrot	*Neophema splendida*	9	3	4
red-crowned parakeet	*Cyanoramphus novaezelandiae*	7	2	3
eastern rosella	*Platycercus eximius*	4	0	1
crimson rosella	*Platycercus elegans*	3	0	1
Fischer’s lovebird	*Agapornis fischeri*	8	2	1
rosy-faced lovebird	*Agapornis roseicollis*	13	2	5
budgerigar	*Melopsittacus undulatus*	43	3	17
	Total	210	50	59

1 PaBV – parrot bornavirus; BFDV – beak and feather disease virus; ApyV – avian polyomavirus

### Infection patterns within bird source sites

**Fig. 3.**
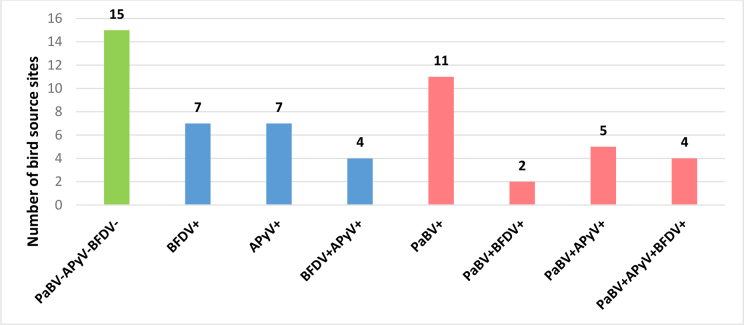
Results of viral testing at the bird source–site level, showing sites free of detectable infections (green), those with single or multiple infections without PaBV (blue) and those with infections involving PaBV (pink). PaBV – psittacine bornavirus; BFDV – beak and feather disease virus; APyV – avian polyomavirus

The necropsied parrots originated from 55 aviaries or households. Viral infections were detected in 72.7% of these locations and PaBV infections in 40%. Mixed infections were more frequently observed in samples from birds from larger breeding aviaries, whereas most samples from parrots from households revealed single infections or the absence of detectable viral pathogens. Detailed data are shown in Fig. 3.

## Discussion

This study provides the first long-term overview of PaBV, BFDV and APyV occurrence in necropsy samples from parrots in Poland. Viral infections were widespread, with more than half of the examined birds testing positive for at least one pathogen. Comparable studies in other regions have reported significant BFDV detection rates. In the Czech Republic, BFDV was detected in 21.5% of clinically healthy captive parrots (23), while surveys in Germany reported BFDV rates exceeding 40% in certain captive populations (15), indicating endemic circulation in European collections. A similar pattern has been observed in captive South American parrots, with BFDV detected in 23.2%. The literature carries dramatically lower APvY prevalence rates than the 31.0% of the present investigation. It occurred in 1.6% of South American and 1.1% of Czech captive psittacines (4, 23); however, those investigations were on samples from all members of a captive population and birds which were clinically healthy, respectively. The sample which yielded our prevalence rate may have had heavy overrepresentation of diseased birds, because each individual was directed to the necropsy series we conducted. Very high rates of PaBV infection (up to 73.7%) were reported in Brazilian aviaries (20), which exceeded nearly threefold our 28.1%. A recent global meta-analysis estimated an overall molecular prevalence of BFDV around 16% (25), further supporting widespread viral presence in psittacine populations worldwide.

In Poland, studies have already been carried out on the occurrence of bornaviruses in necropsy samples of Anseriformes, in which aquatic bird bornavirus was found in only 10% of swans (*Cygnus olor*) (22), and of Atlantic canaries (*Serinus canaria*), in which canary bornavirus was found in 10.2% (21). Compared to these data, the result in the studied parrots is over twice as high because PaBV was detected in nearly one-quarter of the examined parrots, with the highest positivity rates observed in macaws, amazons and caiques. The overwhelming dominance of PaBV-4 mirrors findings from neighbouring European countries (18, 24), indicating that this genotype is well established in captive birds and may represent the dominant circulating strain in Central Europe. According to some authors, grey parrots, macaws, Amazon parrots, conures and cockatoos are the most commonly affected psittacine species (19). The results of our study add weight to this opinion, especially with regard to macaws and Amazon parrots; however, it must be borne in mind that the numbers of grey parrots, conures and cockatoos in our study were low.

The phylogenetic analysis revealed multiple well-defined PaBV-4 subclusters, often corresponding to individual source sites, which strongly suggests intrasite transmission. The presence of clusters containing sequences from different owners and different years points toward inter-site spread, most likely driven by exchange, purchase or movement of birds. However, one important limitation of the phylogenetic analysis should be stated – only partial N-gene sequences were included in phylogenetic analysis, not whole genomes. This may reduce the resolution and accuracy of the inferred relationships.

An important finding is that only a minority of PaBV-positive parrots exhibited gross lesions typical of PDD, consistent with previous studies demonstrating that many PaBV infections remain subclinical (20). This study, which analysed naturally deceased birds submitted for necropsy, provides further evidence of subclinical PaBV infection in this population. The factors determining whether infection progresses to clinical PDD were not investigated here, but likely involve complex interactions between host, viral and environmental factors, as suggested elsewhere (17). Beak and feather disease virus and APyV were more prevalent than PaBV, particularly in small psittacines such as budgerigars and lovebirds. This is in line with previous studies demonstrating that both viruses are highly established in small psittacine species (13, 15). Mixed infections involving BFDV and APyV were frequent, underscoring the complexity of viral epidemiology in captive parrots. Larger breeder aviaries exhibited a higher burden of multi-pathogen infections, consistent with increased stocking density and higher transmission pressure. These findings highlight the importance of aviary management practices, including hygiene, quarantine protocols and routine testing when introducing new birds.

Overall, the phylogenetic and epidemiological findings indicate that PaBV-4, BFDV and APyV circulate widely among parrots in Poland. In this study’s necropsies, PaBV infections were detected in the absence of gross lesions typical of PDD, indicating that infection can be noted without visible pathological changes. This raises concerns about silent viral spread. Routine screening, improved biosecurity and education of bird owners are essential steps toward reducing viral transmission and improving the health and welfare of captive psittacine populations.

## Conclusion

The present study provides the first comprehensive, long-term assessment of PaBV, BFDV and APyV occurrence in parrots in Poland. Our findings demonstrate that all three viruses are widespread, with APyV and BFDV detected more frequently than PaBV. Despite a relatively high prevalence of PaBV, only a minority of infected parrots exhibited macroscopic PDD lesions, and the disproportionality between virus detection and viral lesions shows the complex and possibly subclinical nature of bornavirus infections. Species- and aviary-level differences in infection patterns underscore the importance of routine viral screening and biosecurity measures in both breeder and household settings. Continued monitoring and owner education are essential to limit viral spread and improve the health and welfare of captive parrots in Poland.

## Supplementary Material

Supplementary Material Details
