# Supplementary material for: Occurrence of bornaviruses, circoviruses and polyomaviruses in necropsy samples from parrots in Poland (2014–2024)
Source: J Vet Res. 2026 Mar 25;70(1):1–7. doi: 10.2478/jvetres-2026-0011 (PMC13054757; doi:10.2478/jvetres-2026-0011)
Supplement: Supplementary file 1 — Supplementary Material Details [file jvetres-2026-0011_sm.pdf]

Supplementary Table 1. Similarity % of the analysed nucleotide sequences of *Taenia hydatigena* (776 bp) and the *Taenia lynciscapreoli* sequence (MK033479) used in the phylogenetic tree as an outgroup, according to GenBank accession numbers. The BLASTR NCBI program (https://blast.ncbi.nlm.nih.gov/Blast.cgi) was used for comparison

Strain		1 PQ157678	2 PQ157679	3 PQ157680	4 PQ157682	5 PQ157683	6 PQ157684	7 PQ157685	8 PQ525696	9 PQ525700	10 PP387600	11 PP387611	12 PP387612	13 PP408284	14 MF630925	15 PP387598	16 PP408288	17 PP408290	18 PP408292	19 OR711634	20 OR711638	21 OR830598	22 OQ317804	23 MF630924	24 PP408283	25 PP408293	26 PP387599	27 PP387619	28 PP387620	29 PP408287	30 OR711635	31 PP408282	32 PP408285	33 PP408286	34 OR711637	35 PP408289	36 PP408294	37 OR711631	38 OR711632	39 PP408295	40 MT784872	41 OR830596	42 OR830599	43 PP387596	44 MK945749	45 OQ317833	46 OR830597	47 PP408291	48 OR711633	49 MK033479
1	PQ157678	100.00	99.48	99.36	99.61	99.74	99.74	99.87	99.36	99.61	98.84	98.84	99.23	99.36	99.10	99.61	99.36	99.61	99.61	99.36	99.61	99.48	99.48	99.48	99.48	99.48	99.74	99.23	99.36	99.36	99.48	99.61	99.61	99.48	99.61	99.48	99.61	99.61	99.61	99.74	99.74	99.48	99.74	99.61	99.74	99.74	99.48	99.10	99.10	93.56
2	PQ157679	99.48	100.00	99.36	99.61	99.48	99.48	99.61	99.36	99.61	98.84	98.84	99.23	99.36	99.10	99.61	99.36	99.61	99.61	99.61	99.61	99.48	99.48	99.48	99.48	99.48	99.74	99.23	99.36	99.36	99.48	99.61	99.61	99.48	99.61	99.48	99.61	99.61	99.61	99.48	99.48	99.23	99.48	99.61	99.74	99.74	99.48	99.10	99.10	93.81
3	PQ157680	99.36	99.36	100.00	99.74	99.61	99.61	99.48	99.48	99.48	98.97	98.97	99.36	99.48	99.23	99.74	99.48	99.74	99.74	99.48	99.74	99.61	99.61	99.61	99.61	99.61	99.61	99.10	99.23	99.23	99.36	99.48	99.48	99.36	99.48	99.36	99.48	99.48	99.48	99.36	99.36	99.10	99.36	99.48	99.61	99.61	99.36	99.23	99.23	93.69
4	PQ157682	99.61	99.61	99.74	100.00	99.87	99.87	99.74	99.74	99.74	99.36	99.36	99.61	99.74	99.48	100.00	99.74	100.00	100.00	99.74	100.00	99.87	99.87	99.87	99.87	99.87	99.87	99.36	99.48	99.48	99.61	99.74	99.74	99.61	99.74	99.61	99.74	99.74	99.74	99.61	99.61	99.36	99.61	99.74	99.87	99.87	99.61	99.48	99.48	93.94
5	PQ157683	99.74	99.48	99.61	99.87	100.00	100.00	99.87	99.61	99.61	99.10	99.10	99.48	99.61	99.36	99.87	99.61	99.87	99.87	99.61	99.87	99.74	99.74	99.74	99.74	99.74	99.74	99.23	99.36	99.36	99.48	99.61	99.61	99.48	99.61	99.48	99.61	99.61	99.61	99.74	99.74	99.48	99.74	99.61	99.74	99.74	99.48	99.36	99.36	93.81
6	PQ157684	99.74	99.48	99.61	99.87	100.00	100.00	99.87	99.61	99.61	99.10	99.10	99.48	99.61	99.36	99.87	99.61	99.87	99.87	99.61	99.87	99.74	99.74	99.74	99.74	99.74	99.74	99.23	99.36	99.36	99.48	99.61	99.61	99.48	99.61	99.48	99.61	99.61	99.61	99.74	99.74	99.48	99.74	99.61	99.74	99.74	99.48	99.36	99.36	93.81
7	PQ157685	99.87	99.61	99.48	99.74	99.87	99.87	100.00	99.48	99.74	98.97	98.97	99.36	99.48	99.23	99.74	99.48	99.74	99.74	99.48	99.74	99.61	99.61	99.61	99.61	99.61	99.87	99.36	99.48	99.48	99.61	99.74	99.74	99.61	99.74	99.61	99.74	99.74	99.74	99.87	99.87	99.61	99.87	99.74	99.87	99.87	99.61	99.23	99.23	93.69
8	PQ525696	99.36	99.36	99.48	99.74	99.61	99.61	99.48	100.00	99.48	98.97	98.97	99.36	99.48	99.23	99.74	99.48	99.74	99.74	99.48	99.74	99.61	99.61	99.61	99.61	99.61	99.61	99.10	99.23	99.23	99.36	99.48	99.48	99.36	99.48	99.36	99.48	99.48	99.48	99.36	99.36	99.23	99.36	99.48	99.61	99.61	99.36	99.23	99.23	93.94
9	PQ525700	99.61	99.61	99.48	99.74	99.61	99.61	99.74	99.48	100.00	98.97	98.97	99.36	99.48	99.23	99.74	99.48	99.74	99.74	99.48	99.74	99.61	99.61	99.61	99.61	99.61	99.87	99.36	99.48	99.48	99.61	99.74	99.74	99.61	99.74	99.61	99.74	99.74	99.74	99.61	99.61	99.36	99.61	99.74	99.87	99.87	99.87	99.23	99.23	93.69
10	PP387600	98.84	98.84	98.97	99.23	99.10	99.10	98.97	98.97	98.97	100.00	100.00	99.10	99.23	99.10	99.23	98.97	99.23	99.23	98.97	99.23	99.10	99.10	99.23	99.10	99.10	99.10	98.58	98.71	98.71	98.84	98.97	98.97	98.84	98.97	98.84	99.23	99.23	99.23	98.84	98.84	98.58	98.84	98.97	99.10	99.10	98.84	98.97	98.71	93.81
11	PP387611	98.84	98.84	98.97	99.23	99.10	99.10	98.97	98.97	98.97	100.00	100.00	99.10	99.23	99.10	99.23	98.97	99.23	99.23	98.97	99.23	99.10	99.10	99.23	99.10	99.10	99.10	98.58	98.71	98.71	98.84	98.97	98.97	98.84	98.97	98.84	99.23	99.23	99.23	98.84	98.84	98.58	98.84	98.97	99.10	99.10	98.84	98.97	98.71	93.81
12	PP387612	99.23	99.23	99.36	99.61	99.48	99.48	99.36	99.36	99.36	99.10	99.10	100.00	99.61	99.36	99.61	99.36	99.61	99.61	99.36	99.61	99.48	99.48	99.48	99.48	99.48	99.48	98.97	99.10	99.10	99.23	99.36	99.36	99.23	99.36	99.23	99.36	99.36	99.36	99.23	99.23	98.97	99.23	99.36	99.48	99.48	99.23	99.10	99.10	93.56
13	PP408284	99.36	99.36	99.48	99.74	99.61	99.61	99.48	99.48	99.48	99.23	99.23	99.61	100.00	99.48	99.74	99.48	99.74	99.74	99.48	99.74	99.61	99.61	99.61	99.61	99.61	99.61	99.10	99.23	99.23	99.36	99.48	99.48	99.36	99.48	99.36	99.48	99.48	99.48	99.36	99.36	99.10	99.36	99.48	99.61	99.61	99.36	99.23	99.23	93.81
14	MF630925	99.10	99.10	99.23	99.48	99.36	99.36	99.23	99.23	99.23	99.10	99.10	99.36	99.48	100.00	99.48	99.23	99.48	99.48	99.23	99.48	99.36	99.36	99.61	99.36	99.36	99.36	98.84	98.97	98.97	99.10	99.36	99.23	99.10	99.23	99.10	99.23	99.23	99.23	99.10	99.10	98.84	99.10	99.23	99.36	99.36	99.10	98.97	99.23	94.07
15	PP387598	99.61	99.61	99.74	100.00	99.87	99.87	99.74	99.74	99.74	99.23	99.23	99.61	99.74	99.48	100.00	99.74	100.00	100.00	99.74	100.00	99.87	99.87	99.87	99.87	99.87	99.87	99.36	99.48	99.48	99.61	99.74	99.74	99.61	99.74	99.61	99.74	99.74	99.74	99.61	99.61	99.36	99.61	99.74	99.87	99.87	99.61	99.48	99.48	93.94
16	PP408288	99.36	99.36	99.48	99.74	99.61	99.61	99.48	99.48	99.48	98.97	98.97	99.36	99.48	99.23	99.74	100.00	99.74	99.74	99.48	99.74	99.61	99.61	99.61	99.61	99.61	99.61	99.10	99.23	99.23	99.36	99.48	99.48	99.36	99.48	99.36	99.48	99.48	99.48	99.36	99.36	99.10	99.36	99.48	99.61	99.61	99.36	99.23	99.23	93.69
17	PP408290	99.61	99.61	99.74	100.00	99.87	99.87	99.74	99.74	99.74	99.23	99.23	99.61	99.74	99.48	100.00	99.74	100.00	100.00	99.74	100.00	99.87	99.87	99.87	99.87	99.87	99.87	99.36	99.48	99.48	99.61	99.74	99.74	99.61	99.74	99.61	99.74	99.74	99.74	99.61	99.61	99.36	99.61	99.74	99.87	99.87	99.61	99.48	99.48	93.94
18	PP408292	99.61	99.61	99.74	100.00	99.87	99.87	99.74	99.74	99.74	99.23	99.23	99.61	99.74	99.48	100.00	99.74	100.00	100.00	99.74	100.00	99.87	99.87	99.87	99.87	99.87	99.87	99.36	99.48	99.48	99.61	99.74	99.74	99.61	99.74	99.61	99.74	99.74	99.74	99.61	99.61	99.36	99.61	99.74	99.87	99.87	99.61	99.48	99.48	93.94
19	OR711634	99.36	99.61	99.48	99.74	99.61	99.61	99.48	99.48	99.48	98.97	98.97	99.36	99.48	99.23	99.74	99.48	99.74	99.74	100.00	99.74	99.61	99.61	99.61	99.61	99.61	99.61	99.10	99.23	99.23	99.36	99.48	99.48	99.36	99.48	99.36	99.48	99.48	99.48	99.36	99.36	99.10	99.36	99.48	99.61	99.61	99.36	99.23	99.23	94.20
20	OR711638	99.61	99.61	99.74	100.00	99.87	99.87	99.74	99.74	99.74	99.23	99.23	99.61	99.74	99.48	100.00	99.74	100.00	100.00	99.74	100.00	99.87	99.87	99.87	99.87	99.87	99.87	99.36	99.48	99.48	99.61	99.74	99.74	99.61	99.74	99.61	99.74	99.74	99.74	99.61	99.61	99.36	99.61	99.74	99.87	99.87	99.61	99.48	99.48	93.94
21	OR830598	99.48	99.48	99.61	99.87	99.74	99.74	99.61	99.61	99.61	99.10	99.10	99.48	99.61	99.36	99.87	99.61	99.87	99.87	99.61	99.87	100.00	99.74	99.74	99.74	99.74	99.74	99.23	99.36	99.36	99.48	99.61	99.61	99.48	99.61	99.48	99.61	99.61	99.61	99.48	99.48	99.23	99.48	99.61	99.74	99.74	99.48	99.36	99.36	93.81
22	OQ317804	99.48	99.48	99.61	99.87	99.74	99.74	99.61	99.61	99.61	99.10	99.10	99.48	99.61	99.36	99.87	99.61	99.87	99.87	99.61	99.87	99.74	100.00	99.74	99.74	99.74	99.74	99.23	99.36	99.36	99.48	99.61	99.61	99.48	99.61	99.48	99.61	99.61	99.61	99.48	99.48	99.23	99.48	99.61	99.74	99.74	99.48	99.36	99.36	93.81
23	MF630924	99.48	99.48	99.61	99.87	99.74	99.74	99.61	99.61	99.61	99.23	99.23	99.48	99.61	99.61	99.87	99.61	99.87	99.87	99.61	99.87	99.74	99.74	100.00	99.74	99.74	99.74	99.23	99.36	99.36	99.48	99.61	99.61	99.48	99.61	99.48	99.61	99.61	99.61	99.48	99.48	99.23	99.48	99.61	99.74	99.74	99.48	99.36	99.36	94.07
24	PP408283	99.48	99.48	99.61	99.87	99.74	99.74	99.61	99.61	99.61	99.10	99.10	99.48	99.61	99.36	99.87	99.61	99.87	99.87	99.61	99.87	99.74	99.74	99.74	100.00	100.00	99.74	99.23	99.36	99.36	99.48	99.61	99.61	99.48	99.61	99.48	99.61	99.61	99.61	99.48	99.48	99.23	99.48	99.61	99.74	99.74	99.48	99.36	99.36	93.81
25	PP408293	99.48	99.48	99.61	99.87	99.74	99.74	99.61	99.61	99.61	99.10	99.10	99.48	99.61	99.36	99.87	99.61	99.87	99.87	99.61	99.87	99.74	99.74	99.74	100.00	100.00	99.74	99.23	99.36	99.36	99.48	99.61	99.61	99.48	99.61	99.48	99.61	99.61	99.61	99.48	99.48	99.23	99.48	99.61	99.74	99.74	99.48	99.36	99.36	93.81
26	PP387599	99.74	99.74	99.61	99.87	99.74	99.74	99.87	99.61	99.87	99.10	99.10	99.48	99.61	99.36	99.87	99.61	99.87	99.87	99.61	99.87	99.74	99.74	99.74	99.74	99.74	100.00	99.48	99.61	99.61	99.74	99.87	99.87	99.74	99.87	99.74	99.87	99.87	99.87	99.74	99.74	99.48	99.74	99.87	100.00	100.00	99.74	99.36	99.36	93.81
27	PP387619	99.23	99.23	99.10	99.36	99.23	99.23	99.36	99.10	99.36	98.58	98.58	98.97	99.10	98.84	99.36	99.10	99.36	99.36	99.10	99.36	99.23	99.23	99.23	99.23	99.23	99.48	100.00	99.10	99.10	99.23	99.36	99.36	99.23	99.36	99.23	99.36	99.36	99.36	99.23	99.23	98.97	99.23	99.36	99.48	99.48	99.23	98.84	98.84	93.56
28	PP387620	99.36	99.36	99.23	99.48	99.36	99.36	99.48	99.23	99.48	98.71	98.71	99.10	99.23	98.97	99.48	99.23	99.48	99.48	99.23	99.48	99.36	99.36	99.36	99.36	99.36	99.61	99.10	100.00	99.48	99.61	99.48	99.48	99.36	99.48	99.36	99.48	99.48	99.48	99.36	99.36	99.10	99.36	99.48	99.61	99.61	99.36	98.97	98.97	93.94
29	PP408287	99.36	99.36	99.23	99.48	99.36	99.36	99.48	99.23	99.48	98.71	98.71	99.10	99.23	98.97	99.48	99.23	99.48	99.48	99.23	99.48	99.36	99.36	99.36	99.36	99.36	99.61	99.10	99.48	100.00	99.61	99.48	99.48	99.36	99.48	99.36	99.48	99.48	99.48	99.36	99.36	99.10	99.36	99.48	99.61	99.61	99.36	98.97	99.23	93.94
30	OR711635	99.48	99.48	99.36	99.61	99.48	99.48	99.61	99.36	99.61	98.84	98.84	99.23	99.36	99.10	99.61	99.36	99.61	99.61	99.36	99.61	99.48	99.48	99.48	99.48	99.48	99.74	99.23	99.61	99.61	100.00	99.61	99.61	99.48	99.61	99.48	99.61	99.61	99.61	99.48	99.48	99.23	99.48	99.61	99.74	99.74	99.48	99.10	99.10	94.07
31	PP408282	99.61	99.61	99.48	99.74	99.61	99.61	99.74	99.48	99.74	98.97	98.97	99.36	99.48	99.36	99.74	99.48	99.74	99.74	99.48	99.74	99.61	99.61	99.61	99.61	99.61	99.87	99.36	99.48	99.48	99.61	100.00	99.74	99.61	99.74	99.61	99.74	99.74	99.74	99.61	99.61	99.36	99.61	99.74	99.87	99.87	99.61	99.23	99.23	93.94
32	PP408285	99.61	99.61	99.48	99.74	99.61	99.61	99.74	99.48	99.74	98.97	98.97	99.36	99.48	99.23	99.74	99.48	99.74	99.74	99.48	99.74	99.61	99.61	99.61	99.61	99.61	99.87	99.36	99.48	99.48	99.61	99.74	100.00	99.61	99.74	99.61	99.74	99.74	99.74	99.61	99.61	99.36	99.61	99.74	99.87	99.87	99.61	99.23	99.23	93.69
33	PP408286	99.48	99.48	99.36	99.61	99.48	99.48	99.61	99.36	99.61	98.84	98.84	99.23	99.36	99.10	99.61	99.36	99.61	99.61	99.36	99.61	99.48	99.48	99.48	99.48	99.48	99.74	99.23	99.36	99.36	99.48	99.61	99.61	100.00	99.87	99.48	99.61	99.61	99.61	99.48	99.48	99.23	99.48	99.61	99.74	99.74	99.48	99.10	99.10	93.81
34	OR711637	99.61	99.61	99.48	99.74	99.61	99.61	99.74	99.48	99.74	98.97	98.97	99.36	99.48	99.23	99.74	99.48	99.74	99.74	99.48	99.74	99.61	99.61	99.61	99.61	99.61	99.87	99.36	99.48	99.48	99.61	99.74	99.74	99.87	100.00	99.61	99.74	99.74	99.74	99.61	99.61	99.36	99.61	99.74	99.87	99.87	99.61	99.23	99.23	93.94
35	PP408289	99.48	99.48	99.36	99.61	99.48	99.48	99.61	99.61	99.36	98.84	98.84	99.23	99.36	99.10	99.61	99.36	99.61	99.61	99.36	99.61	99.48	99.48	99.48	99.48	99.48	99.74	99.23	99.36	99.36	99.48	99.61	99.61	99.48	99.61	100.00	99.61	99.61	99.61	99.48	99.48	99.23	99.48	99.61	99.74	99.74	99.48	99.10	99.10	93.56
36	PP408294	99.61	99.61	99.48	99.74	99.61	99.61	99.74	99.48	99.74	99.23	99.23	99.36	99.48	99.23	99.74	99.48	99.74	99.74	99.48	99.74	99.61	99.61	99.61	99.61	99.61	99.87	99.36	99.48	99.48	99.61	99.74	99.74	99.61	99.74	99.61	100.00	100.00	100.00	99.61	99.61	99.36	99.61	99.74	99.87	99.87	99.61	99.23	99.23	93.94
37	OR711631	99.61	99.61	99.48	99.74	99.61	99.61	99.74	99.48	99.74	99.23	99.23	99.36	99.48	99.23	99.74	99.48	99.74	99.74	99.48	99.74	99.61	99.61	99.61	99.61	99.61	99.87	99.36	99.48	99.48	99.61	99.74	99.74	99.61	99.74	99.61	100.00	100.00	100.00	99.61	99.61	99.36	99.61	99.74	99.87	99.87	99.61	99.23	99.23	93.94
38	OR711632	99.61	99.61	99.48	99.74	99.61	99.61	99.74	99.48	99.74	99.23	99.23	99.36	99.48	99.23	99.74	99.48	99.74	99.74	99.48	99.74	99.61	99.61	99.61	99.61	99.61	99.87	99.36	99.48	99.48	99.61	99.74	99.74	99.61	99.74	99.61	100.00	100.00	100.00	99.61	99.61	99.36	99.61	99.74	99.87	99.87	99.61	99.23	99.23	93.94
39	PP408295	99.74	99.48	99.36	99.61	99.74	99.74	99.87	99.36	99.61	98.84	98.84	99.23	99.36	99.10	99.61	99.36	99.61	99.61	99.36	99.61	99.48	99.48	99.48	99.48	99.48	99.74	99.23	99.36	99.36	99.48	99.61	99.61	99.48	99.61	99.48	99.61	99.61	99.61	100.00	99.74	99.48	99.74	99.61	99.74	99.74	99.48	99.10	99.10	93.56
40	MT784872	99.74	99.48	99.61	99.36	99.74	99.74	99.87	99.36	99.61	98.84	98.84	99.23	99.36	99.10	99.61	99.36	99.61	99.61	99.36	99.61	99.48	99.48	99.48	99.48	99.48	99.74	99.23	99.36	99.36	99.48	99.61	99.61	99.48	99.61	99.48	99.61	99.61	99.61	99.74	100.00	99.48	99.74	99.61	99.74	99.74	99.48	99.10	99.10	93.56
41	OR830596	99.48	99.23	99.10	99.36	99.48	99.48	99.61	99.23	99.36	98.58	98.58	98.97	99.10	98.84	99.36	99.10	99.36	99.36	99.10	99.36	99.23	99.23	99.23	99.23	99.23	99.48	98.97	99.10	99.10	99.23	99.36	99.36	99.23	99.36	99.23	99.36	99.36	99.36	99.48	99.48	100.00	99.74	99.36	99.48	99.48	99.23	98.84	98.84	93.30
42	OR830599	99.74	99.48	99.36	99.61	99.74	99.74	99.87	99.36	99.61	98.84	98.84	99.23	99.36	99.10	99.61	99.36	99.61	99.61	99.36	99.61	99.48	99.48	99.48	99.48	99.48	99.74	99.23	99.36	99.36	99.48	99.61	99.61	99.48	99.61	99.48	99.61	99.61	99.61	99.74	99.74	99.74	100.00	99.61	99.74	99.74	99.48	99.10	99.10	93.56
43	PP387596	99.61	99.61	99.48	99.74	99.61	99.61	99.74	99.48	99.74	98.97	98.97	99.36	99.48	99.23	99.74	99.48	99.74	99.74	99.48	99.74	99.61	99.61	99.61	99.61	99.61	99.87	99.36	99.48	99.48	99.61	99.74	99.74	99.61	99.74	99.61	99.74	99.74	99.74	99.61	99.61	99.36	99.61	100.00	99.87	99.87	99.61	99.23	99.23	93.69
44	MK945749	99.74	99.74	99.61	99.87	99.74	99.74	99.87	99.61	99.87	99.10	99.10	99.48	99.61	99.36	99.87	99.61	99.87	99.87	99.61	99.87	99.74	99.74	99.74	99.74	99.74	100.00	99.48	99.61	99.61	99.74	99.87	99.87	99.74	99.87	99.74	99.87	99.87	99.87	99.74	99.74	99.48	99.74	99.87	100.00	100.00	99.74	99.36	99.36	93.81
45	OQ317833	99.74	99.74	99.61	99.87	99.74	99.74	99.87	99.61	99.87	99.10	99.10	99.48	99.61	99.36	99.87	99.61	99.87	99.87	99.61	99.87	99.74	99.74	99.74	99.74	99.74	100.00	99.48	99.61	99.61	99.74	99.87	99.87	99.74	99.87	99.74	99.87	99.87	99.87	99.74	99.74	99.48	99.74	99.87	100.00	100.00	99.74	99.36	99.36	93.81
46	OR830597	99.48	99.48	99.36	99.61	99.48	99.48	99.61	99.36	99.87	98.84	98.84	99.23	99.36	99.10	99.61	99.36	99.61	99.61	99.36	99.61	99.48	99.48	99.48	99.48	99.48	99.74	99.23	99.36	99.36	99.48	99.61	99.61	99.48	99.61	99.48	99.61	99.61	99.61	99.48	99.48	99.23	99.48	99.61	99.74	99.74	100.00	99.10	99.10	93.56
47	PP408291	99.10	99.10	99.23	99.48	99.36	99.36	99.23	99.23	99.23	98.97	98.97	99.10	99.23	98.97	99.48	99.23	99.48	99.48	99.23	99.48	99.36	99.36	99.36	99.36	99.36	99.36	98.84	98.97	98.97	99.10	99.23	99.23	99.10	99.23	99.10	99.23	99.23	99.23	99.10	99.10	98.84	99.10	99.23	99.36	99.36	99.10	100.00	99.23	94.07
48	OR711633	99.10	99.10	99.23	99.48	99.36	99.36	99.23	99.23	99.23	98.71	98.71	99.10	99.23	99.23	99.48	99.23	99.48	99.48	99.23	99.48	99.36	99.36	99.36	99.36	99.36	99.36	98.84	98.97	99.23	99.10	99.23	99.23	99.10	99.23	99.10	99.23	99.23	99.23	99.10	99.10	98.84	99.10	99.23	99.36	99.36	99.10	99.23	100.00	94.20
49	MK033479	93.56	93.81	93.69	93.94	93.81	93.81	93.69	93.94	93.69	93.81	93.81	93.56	93.81	94.07	93.94	93.69	93.94	93.94	94.20	93.94	93.81	93.81	94.07	93.81	93.81	93.81	93.56	93.94	93.94	94.07	93.94	93.69	93.81	93.94	93.56	93.94	93.94	93.94	93.56	93.56	93.30	93.56	93.69	93.81	93.81	93.56	94.07	94.20	100.00
Strain		1 PQ157678	2 PQ157679	3 PQ157680	4 PQ157682	5 PQ157683	6 PQ157684	7 PQ157685	8 PQ525696	9 PQ525700	10 PP387600	11 PP387611	12 PP387612	13 PP408284	14 MF630925	15 PP387598	16 PP408288	17 PP408290	18 PP408292	19 OR711634	20 OR711638	21 OR830598	22 OQ317804	23 MF630924	24 PP408283	25 PP408293	26 PP387599	27 PP387619	28 PP387620	29 PP408287	30 OR711635	31 PP408282	32 PP408285	33 PP408286	34 OR711637	35 PP408289	36 PP408294	37 OR711631	38 OR711632	39 PP408295	40 MT784872	41 OR830596	42 OR830599	43 PP387596	44 MK945749	45 OQ317833	46 OR830597	47 PP408291	48 OR711633	49 MK033479
